# Variant CJD: Reflections a Quarter of a Century on

**DOI:** 10.3390/pathogens10111413

**Published:** 2021-10-30

**Authors:** Diane L. Ritchie, Alexander H. Peden, Marcelo A. Barria

**Affiliations:** National CJD Research & Surveillance Unit, Centre for Clinical Brain Sciences, University of Edinburgh, Western General Hospital, Crewe Road, Edinburgh EH4 2XU, UK; a.peden@ed.ac.uk (A.H.P.); marcelo.barria@ed.ac.uk (M.A.B.)

**Keywords:** variant Creutzfeldt-Jakob disease, prion diseases, BSE, transmission, blood transfusion, prevalence

## Abstract

Twenty-five years has now passed since variant Creutzfeldt-Jakob disease (vCJD) was first described in the United Kingdom (UK). Early epidemiological, neuropathological and biochemical investigations suggested that vCJD represented a new zoonotic form of human prion disease resulting from dietary exposure to the bovine spongiform encephalopathy (BSE) agent. This hypothesis has since been confirmed though a large body of experimental evidence, predominantly using animal models of the disease. Today, the clinical, pathological and biochemical phenotype of vCJD is well characterized and demonstrates a unique and remarkably consistent pattern between individual cases when compared to other human prion diseases. While the numbers of vCJD cases remain reassuringly low, with 178 primary vCJD cases reported in the UK and a further 54 reported worldwide, concerns remain over the possible appearance of new vCJD cases in other genetic cohorts and the numbers of asymptomatic individuals in the population harboring vCJD infectivity. This review will provide a historical perspective on vCJD, examining the origins of this acquired prion disease and its association with BSE. We will investigate the epidemiology of the disease along with the unique clinicopathological and biochemical phenotype associated with vCJD cases. Additionally, this review will examine the impact vCJD has had on public health in the UK and the ongoing concerns raised by this rare group of disorders.

## 1. Introduction

Prion diseases or transmissible spongiform encephalopathies (TSEs) are a group of rare neurodegenerative conditions that occur naturally in humans as well as a range of animal species. Human prion diseases share many clinical and neuropathological features with that of more common neurodegenerative conditions such as Alzheimer’s disease and Parkinson’s disease, in particular the accumulation in the brain of abnormal, disease-specific protein aggregates [1]. In prion diseases, this protein is a misfolded and partially protease-resistant form of a normal host-encoded cellular protein, the prion protein (PrP^C^) [2,3,4]. Consistent with most neurodegenerative conditions, prion diseases can occur in both sporadic and genetic forms; indeed, sporadic Creutzfeldt-Jakob disease (sCJD) is the most common form of human prion disease, accounting for approximately 85% of all human prion diseases [5]. However, a unique feature of prion disease, and one that continues to set them apart from all other neurodegenerative conditions, is that they may also be acquired, with the ability to transmit infectivity between individuals of the same species and in some instances between different species [6]. As a consequence of this infectious nature, prion diseases represent a neurodegenerative condition that poses a serious public health concern.

In 1996, a new form of acquired human prion disease was identified in the United Kingdom (UK). Initially referred to as “new variant” CJD (nvCJD) and since shortened to variant CJD (vCJD) [7], this condition would propel this rare group of neurodegenerative disorders to the forefront of the scientific community and would have an unprecedented and lasting impact on public health in the UK. Twenty-five years on from the first identification of vCJD, this review will provide a historical perspective of this unique form of acquired human prion disease—from its origins and first recognition in humans, to current diagnosis and epidemiology of the disease. Additionally, we will detail the public health impact following the identification of vCJD in the UK and the current public health concerns that still surround this condition a quarter of a century on.

## 2. The Emergence of vCJD

**Figure 1:**
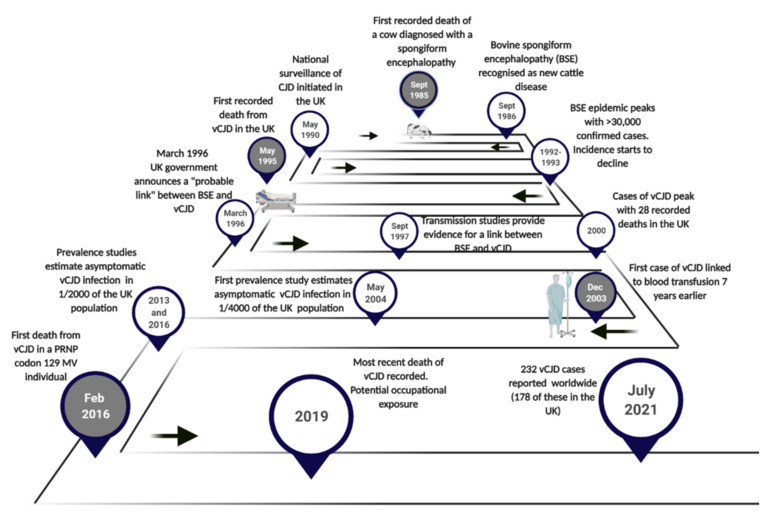
Timeline of the last 35 years highlighting significant dates in the origin, emergence and progression of vCJD. Time points in grey correspond to Western blot images presented in Figure 4d.

The first cases of vCJD were reported in the UK in 1996 (Figure 1) [7]. However, the origins of this human prion disease date back approximately ten years earlier to the emergence of bovine spongiform encephalopathy (BSE) in the UK, a novel prion disease affecting cattle [8]. From its first identification in 1985 (Figure 1), annual numbers of BSE cases in cattle increased rapidly in the UK, reaching a peak in 1992 with more than 37,000 cases recorded (Figure 1) [9]. Subsequently, cases of BSE have been identified in an additional 25 countries, predominantly in Europe, Asia, the Middle East and North America [10]. While the original source of BSE is yet to be clearly established, the epidemic of BSE in cattle has been attributed to the recycling of prion contaminated carcasses (such as sheep with scrapie or a sporadic case of cattle BSE) in the manufacturing of cattle feed in the form of meat and bone meal (MBM) [11,12]. On the other hand, subsequent transmission of atypical forms of BSE in cattle [13,14,15] and scrapie in sheep [16] into transgenic mouse models over-expressing bovine PrP induce strain features similar to that of classical BSE.

From 1988, a number of rigorous control measures were implemented in the UK (and subsequently emulated in some other countries) including (i) the introduction of a ban on the supply and use of ruminant-derived protein in ruminant feed, (ii) the compulsory slaughter and disposal of remains of all cattle suspected of having BSE, (iii) a ban on the feeding of specified bovine offal to humans and farmed animals, (iv) the targeted surveillance and mandatory reporting of clinical neurological disease in cattle, and (v) the removal of cattle over 30 months from the food chain [11,12]. As a result, numbers of BSE cases in the UK declined rapidly, and today BSE is considered a rare disease with only a single case recorded in the UK over the last five years [9]. It is worth noting that the steady decline in BSE cases has led to a relaxation of some of these measures over the last number of years [17].

Inevitably, the emergence of the BSE epidemic in the UK raised public health concerns over the potential cross-species transmission of BSE to humans through dietary exposure to the BSE agent through contaminated food products. These concerns were heightened following parallel reports of a similar spongiform encephalopathy in domestic and captive cat species (now referred to as feline spongiform encephalopathy) and in some species of captive exotic ungulates [18,19,20,21]. The possibility that prion disease in these animals had resulted by the cross-species transmission of BSE through food supplements was thought the most likely cause. In response to these concerns, the UK government reinstated epidemiological surveillance and research into Creutzfeldt-Jakob disease (CJD) in May 1990 (Figure 1) in order to monitor and identify any changes in the incidence or pattern of CJD in the wake of the BSE epidemic [22]. This expansion in surveillance was emulated in a number of other European countries, and following the identification of vCJD in the UK, it was established in other non-European countries as well [23,24,25,26,27,28].

In 1996, a seminal report by Robert Will and colleagues described 10 cases of CJD identified in UK patients of an unusually young age (median 29 years, while sporadic CJD primarily affects individuals over the age of 60) [7]. These 10 patients presented with atypical clinical symptoms and a highly consistent neuropathological phenotype that had not previously been recognised in any other forms of human prion disease. Furthermore, the disease duration was much longer than that observed in patients diagnosed with the more commonly occurring sporadic CJD (sCJD). An alternative diagnosis of iatrogenic CJD (iCJD) was thought unlikely in these patients because investigation of the clinical histories found no evidence of potential iatrogenic exposure to CJD through either dura mater grafting, a history of childhood treatment with pituitary derived growth hormone, neurosurgery or blood transfusion. In addition, genetic sequencing on eight of the 10 cases found no mutations in the prion protein gene (*PRNP*) that is associated with the inherited forms of prion disease. Will and colleagues concluded that these 10 cases represented a new clinicopathological variant of CJD and, having occurred so soon after the BSE epidemic, and with cases exclusively found in the UK (where the BSE epidemic was at its most severe), the most “plausible interpretation” of the findings was that these cases were a direct result of human exposure to the BSE agent [7]. In March 1995, these findings were reported to the UK government by the Spongiform Encephalopathy Advisory Committee (SEAC) of the Department of Health (DOH) and Ministry of Agriculture, Fisheries and Food (MAFF). In addition to public concern over the emergence of this new acquired form of prion disease, the identification of vCJD led to a worldwide ban on the export of British beef causing a severe economic impact in the UK beef industry [29]. Like several other control measures implemented in the wake of the BSE epidemic, the ban posed on the exportation of British beef has since been lifted in Europe, and more recently in the United states [30,31].

## 3. BSE and vCJD, A Single Strain of Agent

A large body of evidence now exists supporting the hypothesis that vCJD has resulted from human infection with the BSE agent. In addition to epidemiological arguments, the most compelling evidence has come from experimental animal models and from analysis of the biochemical phenotype of the misfolded and disease-associated form of the prion protein (PrP^Sc^) that accumulates in the brain and peripheral tissues of vCJD patients. A notable feature of the zoonotic transmission of BSE to other species is the maintenance of its distinctive molecular signature [32]. However, animal transmission studies have recently highlighted the concern that cross-transmission of BSE to sheep or goats may result in a subtle adaptation of BSE, resulting in increased virulence towards humans [33,34].

### 3.1. Animal Models of vCJD

In the same year that vCJD was first described in the UK, results from the intracerebral inoculation of brain isolates from BSE-infected cattle in cynomologus macaques demonstrated that BSE was highly infectious in this primate model [35]. Examination of brain tissue at post-mortem revealed a pattern of pathology in the macaques that was indistinguishable to that observed in vCJD patients, but which differed to that obtained with sCJD in the same primate model [35].

Shortly after this report, a seminal study by Moira Bruce and colleagues published the interim results from the first experimental inoculation of vCJD brain isolates in inbred strains of wild-type mice [36]. These wild-type mouse lines (RIII, VM and C57BL), which carry different alleles of the mouse PrP gene (Prn-p), are a mainstay in prion research, able to discriminate between different prion “strains” based on their unique biological properties on serial passage, specifically by incubation periods and patterns of neuropathological targeting, known as the “lesion profile” [37,38,39,40,41]. Experimental transmission studies carried out during the peak of the BSE epidemic demonstrated a single unique strain “signature” associated with cattle BSE when passaged in these mouse lines [42,43]. Remarkably, this corresponding BSE signature was also observed following transmission of infected brain tissue from other animal species, including domestic cats, greater kudu and Nyala, confirming that the same single strain of agent was responsible for these other animal prion diseases [44,45]. In the study performed by Bruce and colleagues, intracerebral inoculation of brain isolate from three vCJD patients in wild-type mice resulted in transmission properties (incubation period and lesion profile) that were indistinguishable from that of cattle BSE and other BSE-related prion disorders [36]. Crucially, the strain signature differed from the diseased-associated pattern obtained with sCJD brain isolates passaged in the same mouse lines. While the published data was based on interim results from a single mouse strain (RIII), this was considered as a strong indication that the same strain of agent was involved in BSE and in vCJD, and that BSE presented a possible zoonotic risk resulting in vCJD. The complete data set from these three primary transmissions, including immunohistochemical and biochemical analysis of PrP^Sc^ in the brain and subsequent secondary passages, was published several years later, alongside transmission data from an additional seven vCJD patients [46,47]. With the same BSE signature observed in all transmission experiments, this information was taken as convincing evidence that vCJD was a result of human infection with the BSE agent. The studies by Bruce and colleagues have been further supported by a wealth of experimental transmission studies in both wild-type and transgenic mouse models that appear to confirm that BSE in cattle and vCJD in humans are caused by a single agent strain [48,49,50].

In addition to confirming an association with exposure to BSE, animal models have been instrumental over the last 25 years in contributing to our understanding of many other biological aspects of vCJD. Transgenic mouse models have been fundamental in establishing the influence of the naturally occurring methionine (M)/valine (V) polymorphism at codon 129 of *PRNP* on the susceptibility to vCJD [51,52,53,54]. Similarly, wild-type mouse models have highlighted the different pathogenesis in vCJD when compared to other prion diseases, with infectivity detected in lymphoreticular tissues [47,55]. Subsequent studies in both wild-type and transgenic mice demonstrated that infectivity in lymphoreticular tissues may occur in vCJD patients prior to neuroinvasion and the onset of disease [56]. Such properties continue to have a significant impact on public health concerns and the implementation of public health policies. Furthermore, these same mouse studies have confirmed that the BSE/vCJD strain is not generally altered by the tissue of origin [47,55,56], the *PRNP* codon 129 genotype of the individual [56,57,58], geographical location [59,60], age [60] or following secondary human transmission of vCJD through blood transfusion [56,61].

### 3.2. Biochemical Properties of PrP^Sc^ in vCJD

In the absence of any identifiable nucleic acid associated with prion diseases, the phenomenon of prion strains is thought to be enciphered by conformational variability in PrP^Sc^, the misfolded and disease-associated form of the prion protein that accumulates in the brain (and peripheral tissues in vCJD) [62,63,64,65,66,67,68,69]. Such variabilities are commonly measured by examining the molecular weight (N-terminal truncation of the protein) and glycosylation profile (occupancy of the two asparagine-linked glycosylation sites) of PrP^Sc^ after limited proteolytic digestion (PrP^res^) using Western blot analysis [32,63,68]. In the initial analysis of PrP^res^ from brain tissue of vCJD patients, two separate studies, led by John Collinge and Piero Parchi, respectively, described a distinct and consistent PrP^res^ profile that had not been commonly observed in any form of human prion disease, apart from some familial cases [32,69]. This PrP^res^, termed type 2B (Parchi classification) [69] or type 4 (Collinge/London classification) [32] is characterised by an unglycosylated fragment size of ~19kDa and a marked predominance in the diglycosylated fragment of PrP^res^. This PrP^res^ profile is identical to the molecular signature for BSE in cattle and to that of naturally transmitted BSE in domestic cats, and in BSE experimentally transmitted to wild-type mice and macaques, further supporting a causal association between BSE and vCJD [32].

## 4. Epidemiology

### 4.1. Primary Cases of vCJD

As of July 2021, twenty-five years from the first descriptions of vCJD in the UK, 232 clinical cases of definite or probable vCJD have been reported worldwide, in individuals from 12 countries, including the UK (178 cases), France (28 cases), Spain (5 cases), Republic of Ireland (4 cases), U.S.A (4 cases), Italy (3 cases), Netherlands (3 cases), Portugal (2 cases), Canada (2 cases), Taiwan (1 case), Saudia Arabia (1 case) and Japan (1 case) [70]. With vCJD causally linked to the BSE epizootic, it is perhaps unsurprising that the majority of vCJD cases have occurred in the UK where the BSE epidemic was most severe. In non-UK countries, the presence of vCJD has been associated with exposure to the BSE agent through (i) indigenous cases of BSE in cattle within the country of residence [10,71], (ii) UK residency during the period when BSE exposure was at its highest levels [72,73,74,75,76,77], or (iii) through importation of contaminated beef products from the UK [75,78,79].

Cases of vCJD have declined over the last two decades, having reached a peak in 2000 with 29 recorded deaths (28 in the UK and 1 in France) [70]. The last known UK case of vCJD was reported in 2016 with a clinical onset in 2014 [80]. In non-UK vCJD cases, the last case was reported in France in 2019 with the patient having an onset in 2017 (Figure 1) [81]. While dietary exposure to the BSE agent is recognised as the prevailing cause of vCJD cases [82], the last recorded case of vCJD has been associated with possible occupational exposure (i.e., a laboratory accident) [81,83].

Of the 232 vCJD cases who have undergone full genetic analysis, all but a single case has occurred in individuals homozygous for methionine (MM) at codon 129 on *PRNP*, a recognised risk factor for human prion diseases. The remaining vCJD case, and the last reported in the UK, was the first pathologically confirmed case of vCJD in a methionine/valine (MV) heterozygote patient [84]. A suspected case of vCJD in a MV heterozygote had been described in 2009 [85]; however, in the absence of a post-mortem, a diagnosis of vCJD was not confirmed. The identification of vCJD in a *PRNP* codon 129 MV individual was not unexpected because earlier experimental evidence from humanised transgenic mouse models, expressing physiological levels of the human prion protein, indicated that other *PRNP* codon 129 genotypes are susceptible to infection with the BSE agent but may be subject to more prolonged incubation periods [53]. In addition, in vitro amplification experiments using human post-mortem tissue from patients who died from non-neurological conditions and humanised transgenic mouse brains had shown that normal cellular prion protein (PrP^C^) from MV individuals could be converted by MM vCJD PrP^Sc^, albeit with decreased efficiency [86]. While the pathological and biochemical features of vCJD in the MV individual were indistinguishable from that of vCJD in MM individuals, the clinical features were more consistent with those described in sCJD patients [84]. This has raised the possibility that any future cases of vCJD occurring in *PRNP* codon 129 genotypes other than MM may be more difficult to identify based on clinical presentation. This highlights the importance of continued surveillance of human prion diseases in the UK, involving the post-mortem examination of patients [83]. Experimental transmission of brain isolates from the case of vCJD in a MV individual, into a wild-type mouse model, provided evidence that strain properties were identical to those observed for the agent associated with vCJD MM individuals [58]. This observation was consistent with the views that a single unique strain of agent was involved with BSE and vCJD, and it suggests that there is no adaption of this strain in a different genetic background.

### 4.2. Secondary Human-to-Human Transmission of vCJD in the UK

While there is no epidemiological evidence to suggest that sCJD is transmissible via blood transfusion [87,88], the greater extent of peripheral pathogenesis, particularly in lymphoreticular tissues, in vCJD cases [89,90,91,92], raised concerns that vCJD poses a greater risk for iatrogenic secondary transmission, potentially via surgery or blood transfusion. This fear was supported by animal studies demonstrating that BSE in experimentally-infected sheep could be successfully transmitted via transfusion with blood collected during the clinical and the asymptomatic phase of the disease [93,94,95]. In response, and with an increasing number of vCJD cases, a look back study, the Transfusion Medicine Epidemiology Review (TMER), was established in 1997 to look for any evidence that CJD may have transmitted via the UK blood supply.

**Figure 2:**
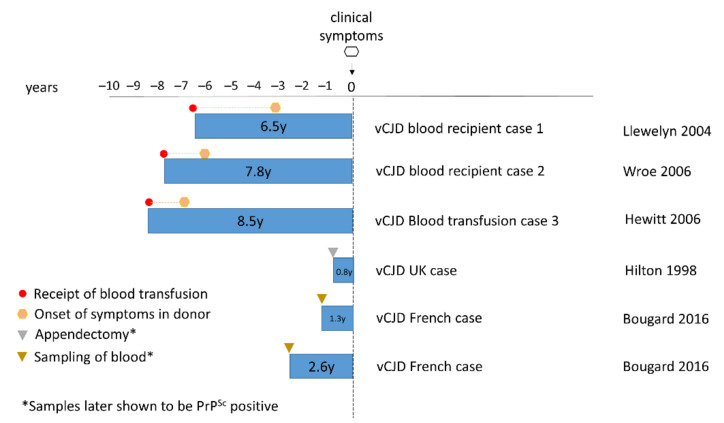
Pre-symptomatic phases for donors and recipients of vCJD-infected blood. Comparison of the lengths of the pre-symptomatic phase of vCJD patients who had received blood from infected donors. For the four cases of vCJD infection following blood transfusion (including a preclinical case), the time elapsed between receiving the blood transfusion (red circles) and the onset of clinical symptoms (or death) is indicated. The time after the donation when clinical onset occurred in the donor is also indicated (orange hexagon).

The above concerns were justified in 2003 with the occurrence of the first case of vCJD following blood transfusion from a donor who later developed vCJD (Figure 1) [96]. Subsequently two further clinical cases of vCJD following blood transfusion were reported [97,98]. All three clinical cases of vCJD following blood transfusion were MM at *PRNP* codon 129. In 2004, a case of preclinical vCJD infection following blood transfusion was reported [99]. Post-mortem examination of this case showed evidence of vCJD PrP^Sc^ deposition in the spleen and cervical lymph node but not in the brain. The patient had died from a condition unrelated to prion disease, five years following blood transfusion from an infected donor [99]. In contrast to the clinical transfusion transmitted vCJD cases, the preclinical case was MV at *PRNP* codon 129. While it is unknown whether they would have gone on to develop vCJD, this was the first demonstration that vCJD infection could replicate in an MV individual. Subsequently, infectivity in the spleen from this case has been confirmed by bioassay [56]. The strain characteristics matched those previously described for vCJD/BSE, demonstrating that replication in lymphoreticular tissue, and in a *PRNP* codon 129 MV genetic background, do not affect the characteristics of the vCJD strain. All four blood transfusion cases suggest that there is sufficient infectivity in blood at a presymptomatic stage to cause infection in another individual. Figure 2 shows that the incubation periods between transfusion and the onset of clinical symptoms varied from 6.5 to 8.5 years. This is broadly in line with incubation period for vCJD estimated from the lag between the peaks of the BSE and vCJD epidemics [100,101,102]. Shown as well is the time interval between the donation of blood and the onset of symptoms in the donor (Figure 2). This is indicative that the vCJD-infected donors potentially may harbour significant infectivity in their blood at least 1.4–3.3 years prior to the appearance of vCJD symptoms.

Each of the four-blood transfusion associated vCJD infections occurred in recipients of non-leucoreduced blood, prior to leucodepletion of all blood components in the UK that was brought into effect in 1999. The TMER study identified 67 individuals who received blood components from vCJD donors, and it is encouraging that there have been no further cases of vCJD following blood transfusion, suggesting that leucodepletion was an effective countermeasure [97,103]. However, animal model experiments demonstrated that all blood components could potentially transmit disease, suggesting a need for continued surveillance for blood transfusion-associated vCJD [104].

Studies of blood donor and recipient pairs suggest that we should not expect any change in the clinical and pathological profile or agent strain as a result of secondary transmission [61,103,105]. However, it needs to be borne in mind that future cases of vCJD following blood transfusion would most likely occur in older individuals as the risk of requiring a transfusion increases with age [106]. In addition, some models of vCJD blood transmission, using both mice and macaques, have identified a different class of neurological impairment. It was demonstrated that these impairments had a prion aetiology but notably lacked classic neuropathological features of prion disease, including deposition of PrP^Sc^ [107]. This suggests the scope of surveillance may need to widen to include neurological conditions that do not fit the current criteria for prion disease and where protease-resistant PrP is absent.

In addition to blood components, another potential means for secondary transmission is human plasma derived medical products. In 1999, a ban on the use of UK sourced plasma for manufacturing blood products that was imposed to prevent secondary vCJD transmission. However, active tissue-based surveillance of patients who had received blood products, identified evidence of vCJD infection in a haemophiliac patient who, many years prior to the aforementioned ban, had received treatment with factor VIII concentrate [108]. Batches of factor VIII concentrate had been prepared by pooling large numbers of UK human plasma donations. Some of the batches received by the patient in question were subsequently found to have been derived from plasma pools that included a donation from a vCJD-infected donor. This haemophiliac patient, who was MV at *PRNP* codon 129, died from causes unrelated to CJD, but PrP^Sc^ was detected in their spleen, suggesting they may have acquired the vCJD prion agent as a result of factor VIII therapy. This same study found no evidence of vCJD infection in autopsy or biopsy material from 16 other haemophiliac patients who had received plasma-derived factor VIII [108]. It should be noted that the ban on the use of UK sourced plasma for blood products was subsequently lifted in 2019 following a report that revised down the risks of future vCJD cases arising through treatment with plasma products [109].

The risk of future clinical cases of secondary transmission vCJD depends on the prevalence of subclinical vCJD in the population and likelihood that subclinical carriers will go onto develop vCJD. The risk of secondary transmission via blood or blood products could be assessed with a rapid and highly sensitive assay that could be used to screen donations. No such assay has yet been fully developed or validated, but several scientific reports have proven the principle that vCJD PrP^Sc^ could be directly captured and detected in blood with a sensitivity of around 70% and very high specificity [110,111].

More recently, developments in the use of in vitro conversion assays, specifically the protein misfolding cyclic amplification (PMCA) assay, have proven sufficiently powerful to amplify and detect PrP^Sc^ in blood or plasma from vCJD patients at both clinical and preclinical stages with near 100% specificity and sensitivity [112,113,114,115]. Similar to PMCA, the real-time quaking-induced conversion (RT-QuIC) assay is another powerful method based on amplifying misfolded PrP [116,117]. RT-QuIC analysis of cerebrospinal fluid is now an important tool for ante-mortem diagnosis and surveillance for sCJD. However, technical challenges remain, specifically with RT-QuIC’s ability to detect vCJD PrP^Sc^ and PrP^Sc^ in blood [118,119,120,121].

## 5. Diagnosis

**Figure 3:**
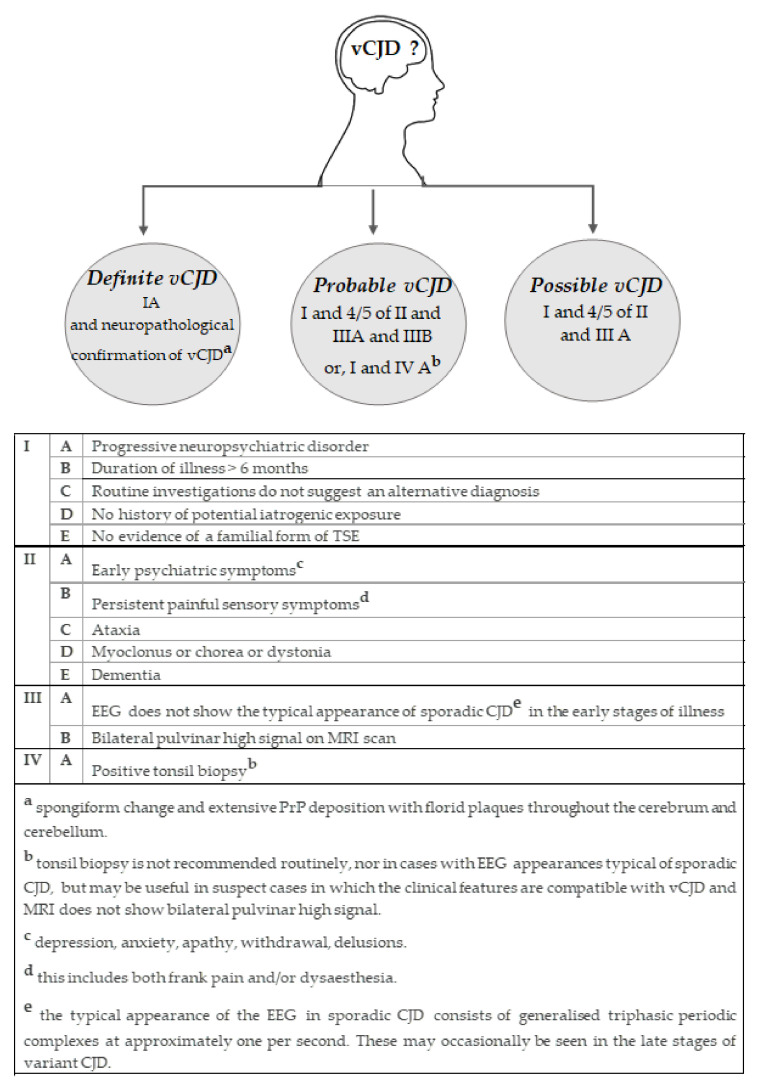
Diagnostic criteria for variant Creutzfeldt-Jakob disease. Adapted from http://www.cjd.ed.ac.uk/sites/default/files/criteria_0.pdf [122] (Accessed on 1 July 2021).

In line with other human prion diseases, internationally agreed diagnostic criteria is available for the diagnosis of vCJD which categorise cases as either “definite”, “probable” or “possible” (Figure 3) [122]. These criteria are based on the acquisition and assessment of clinical, histological, immunohistochemical, biochemical and genetic investigations and has proven highly sensitive and specific in the classification of vCJD cases [123].

### 5.1. Clinical Features

In contrast to other forms of prion disease, vCJD affects individuals of an unusually young age, predominantly in the third decade of life. The youngest vCJD patient, one of two cases reported in Portugal, had a clinical onset of 11 years [124]. The oldest patient had an onset of 74 years; one of only six vCJD patients reported having a disease onset over the age of 55 years [80]. The prevalence of vCJD in this young age distribution is thought to be attributed to age-related differences in either dietary exposure to the BSE agent [125] or in disease susceptibility [126]. Mean duration of illness is approximately 14 months, much longer than the 4 months reported in sCJD cases [80].

The clinical symptoms of vCJD are relatively uniform, predominantly presenting with psychiatric features such as anxiety, withdrawal, depression and personality change [123,127,128]. As a consequence, there are several early accounts of patients having been referred to psychiatric services in the first instance [128]. Psychiatric features may present on their own but can be accompanied by a range of sensory abnormalities that include limb pain and parasthesiae [129]. Neurological signs of disease, typically develop around six months and include; progressive ataxia, visual problems, cognitive decline and involuntary movements including myoclonus, dystonia and chorea [130]. The end stage of disease is similar to that observed in sCJD patients, with terminal akinetic mutism in many cases.

There are a number of additional clinical investigations that have proven valuable in supporting a diagnosis of vCJD and, crucially, in excluding potential treatable conditions. One of the most useful is the magnetic resonance imaging (MRI) brain scan, which produces a distinctive high signal on T2 and fluid-associated inversion recovery (Flair) sequence in the posterior thalamus in vCJD patients. Referred to as the “pulvinar sign”, this forms part of the diagnostic criteria for a possible and probable diagnosis for vCJD, having been observed in over 90% of cases examined (Figure 3) [122,131]. The electroencephalogram (EEG), cerebral spinal fluid (CSF) 14-3-3 assay and RT-QuIC assay, which have proven helpful in the diagnosis of sCJD [132,133,134], are less useful in suspected cases of vCJD. The periodic sharp wave complexes that are characteristic of the EEG in sCJD patients are not a feature in vCJD patients; however, there are some rare reports that these feature in the later stages of the disease (Figure 3) [73,135]. Analysis of CSF 14-3-3 levels shows positivity in only 50% of vCJD cases, compared to around 90% of sCJD cases. However, elevated levels of phospho-tau in the CSF have been reported in many cases of vCJD [136].

### 5.2. Neuropathology

In vCJD, as in all forms of prion disease, neuropathological examination is a requirement for a definite diagnosis. Brain biopsy may be performed in patients where a diagnosis of CJD has been raised; however, this is rare and normally only occurs in cases where a treatable condition is offered in the differential diagnosis. Examination of the brain at autopsy is more common. Macroscopic analysis of the brain in vCJD cases is often unremarkable, but some cerebral and cerebellar atrophy has been described in cases where the disease duration extends over 19 months [137].

**Figure 4:**
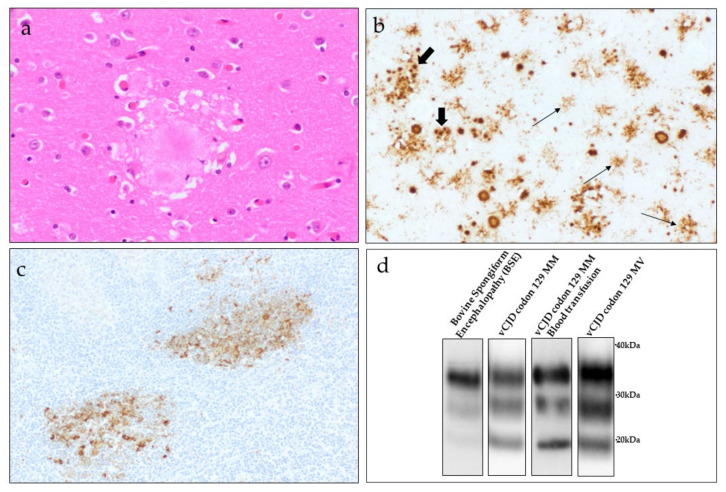
Pathological and biochemical features of vCJD. (**a**) A cluster of large rounded florid plaques present in the cerebral cortex surrounded by a halo of mild spongiform change (Haematoxylin and Eosin stain). Original magnification; ×400 (**b**) Immunohistochemistry for the prion protein shows intense labelling of the florid plaques, and also demonstrates multiple smaller cluster plaques (thick arrows) in addition to pericellular PrP deposits (thin arrows) in the frontal cortex. Original magnification; ×200. (**c**) Peripheral pathology in vCJD demonstrating immunostaining for the prion protein in germinal centres within the tonsil. Original magnification; ×200. (**d**) Representative Western blot analysis of post-mortem brain samples from a case of bovine spongiform encephalopathy in cattle and three individuals with vCJD. Western blot images are compiled from individual Western blot analysis. The approximate positions of molecular mass markers are indicated in kilodaltons.

Histological and immunohistochemical examination has revealed a highly consistent pattern of neuropathology in vCJD cases, very different to that of sCJD where there is marked heterogeneity in terms of pathological phenotype [138]. Spongiform change is a consistent feature in vCJD occurring in a widespread pattern throughout the cerebral and cerebellar cortex, often found in a patchy distribution. The spongiform change is generally most severe within the caudate nucleus and putamen in the basal ganglia. While spongiform change is less prominent in the thalamus, severe neuronal loss and gliosis are a consistent feature in the posterior nuclei, particularly the pulvinar. It is highly likely that this pathology corresponds to the high signal observed on MRI in vCJD patients [131]. The most striking pathological feature of vCJD is the presence of numerous “florid plaques” within the cerebral and cerebellar cortex [137,138,139]. These plaques have the appearance of the classic kuru-type plaques described in cases of kuru and sCJD MV2 cases, comprising a dense eosinophilic amyloid core with radiating peripheral fibrils, but in cases of vCJD, these plaques are often much larger and are surrounded by a halo of spongiform change (Figure 4a) [138]. Florid plaques are rarely observed in other forms of human prion diseases, although occasional florid plaques have been reported in cases of iatrogenic CJD associated with dura mater grafting [140,141,142].

Immunohistochemistry for PrP highlights a distinct and intense pattern of PrP accumulation in the brain of vCJD cases [137,138]. In addition to the large florid plaques, smaller plaque-like aggregates are identified by PrP immunohistochemistry, appearing in small clusters throughout the cerebral cortex (Figure 4b). These plaque-like aggregates are rarely visible through routine histological staining. In addition to plaques, small amorphous ‘feathery’ pericellular deposits of PrP are found surrounding the neurons or astrocytes throughout the grey matter regions of the cerebral and cerebellar cortex. The basal ganglia show a distinct PrP staining pattern in vCJD, comprising multiple small intense punctate deposits occurring in a linear pattern of distribution [138].

### 5.3. Peripheral Pathology

Variant CJD differs from other forms of human prion disease in that there is widespread and readily detectable accumulation of PrP^Sc^ in peripheral tissues of the body, primarily associated with follicular dendritic cells within the follicles of lymphoid tissue (tonsil, spleen, appendix, lymph nodes) (Figure 4c) [89,90]. Immunohistochemistry on archived appendectomy samples from patients that went on to develop clinical vCJD, demonstrated that PrP^Sc^ can accumulate in appendix tissue at least two years prior to the onset of clinical symptoms [91]. Bioassay in wild-type mice has since confirmed infectivity associated with PrP^Sc^ found within spleen and tonsil, but at levels around 100 to 1000 times lower than that demonstrated in the brain [55]. More recently, the detection of infectivity and/or PrP^Sc^ in the spleen of two suspected cases of asymptomatic vCJD compounded concerns over the potential spread of vCJD by iatrogenic methods, particularly in regard to the use of surgical instruments [56,99,108]. The significance of PrP^Sc^ accumulation within lymphoreticular tissue in vCJD is demonstrated by the inclusion of a positive tonsil biopsy becoming part of the diagnostic criteria for vCJD (Figure 3) [90,122].

In addition to lymphoreticular tissues, PrP^Sc^ has also been identified in a range of tissues of the autonomic and peripheral nervous tissues (including; trigeminal, dorsal root and autonomic ganglia) [143], peripheral motor nerve fibres in skeletal muscle [144], pituitary gland [145], and in the dura mater, liver, pancreas, kidney, ovary, uterus and skin in a single vCJD patient with a prolonged disease duration [146].

More recently, the use of the in vitro conversion assay PMCA in the detection of minute quantities of PrP^Sc^ has suggested that the distribution of PrP^Sc^ in both clinical and asymptomatic vCJD cases, and sCJD cases may be more widespread than initially thought [147,148]. While PMCA is not currently included as a diagnostic tool for prion diseases, recent studies have demonstrated that PMCA protocols offer sufficient sensitivity and specificity for the amplification of minute amounts of PrP^Sc^ in biological fluids from vCJD patients including, urine [149], blood [112,113,115] and CSF [150,151]. The detection of PrP^Sc^ in blood was also demonstrated in blood samples taken at the presymptomatic stage of vCJD [112]. No such detection of PrP^Sc^ in blood has been described with other human prion diseases, although PMCA has recently detected PrP^Sc^ in widespread peripheral tissues of sCJD patients [148].

### 5.4. Biochemical Features

Biochemical analysis of frozen tissues (primarily CNS tissue) is a valuable addition in the diagnosis of prion diseases. Western blot analysis of the protease-resistant fragments of the prion protein from the brain of vCJD patients has identified a single PrP^res^ type, termed type 2B (also referred to as type 4 using the less commonly used London classification), associated with vCJD-infected tissues [32,68]. This type 2B PrP^res^ provides a “molecular signature” for vCJD and is characterised by an unglycosylated fragment size of ~19kDa, similar to that observed in sCJD type 2A cases but with a marked predominance in the diglycosylated fragment of PrP^res^ (Figure 4d) [138]. PrP^res^ detected within lymphoretricular tissues in vCJD cases also show this type 2B biochemical phenotype. However, the non-CNS tissues may show a further accentuation in the diglycosylated fragment and an unglycosylated PrP^res^ fragment that may be slightly smaller [89,90].

To date, PrP^res^ type 2B has been identified in the brain of all clinical cases of primary vCJD examined, regardless of the country of origin, or whether the codon 129 *PRNP* genotype of the patient was MM or MV (Figure 4d) [71,75,84,152]. This same PrP^res^ type was also identified in the brain (Figure 4d) and tonsil from transfusion-associated secondary vCJD [98,105] and in the spleen in two cases of asymptomatic vCJD infection [99,105]. This distinctive PrP^res^ type is rarely observed in other forms of human prion diseases.

## 6. Current Public Health Concerns from vCJD

Over the last decade, the numbers of cases of vCJD have declined [70,80]. While this is reassuring, uncertainty surrounding possible future cases of vCJD continues to pose a challenge for public health.

In the UK, concerns over a potential second wave of vCJD in individuals from different *PRNP* codon 129 genotypes resurfaced in 2017, following the first report of pathologically confirmed vCJD in a *PRNP* codon 129 MV patient (Figure 1) [84]. Until 2016, all definite and probable cases of vCJD (with genetic testing) had occurred in *PRNP* codon 129 MM individuals. The appearance of vCJD, the last case reported in the UK, in a *PRNP* codon 129 heterozygous individual, raised the possibility that further vCJD cases may appear in this genotype, but they may be subject to extended incubation periods. This was supported by earlier evidence from experimental transmission studies in humanised transgenic mice (HuMM, HuMV and HuVV), which suggested that all three *PRNP* codon 129 genotypes are susceptible to vCJD infectivity, but that each genotype is subject to differences in the efficiency of transmission [53]. In this transmission study, extended incubation periods were observed in HuMV mice compared to the HuMM mice, and, while clinical disease was not observed in the HuVV mice, the appearance of neuropathology consistent with a prion disease at autopsy indicated that *PRNP* codon 129 VV individuals may also be susceptible to vCJD but after very long incubation times. It is worth noting that the animals in this study, like the majority of animal modelling prion diseases, were challenged via the intracerebral route of inoculation (IC). Whilst IC exposure represents a highly efficient method of prion transmission in mice, results from this study may not provide a true reflection of vCJD in humans resulting from oral exposure to BSE, or in cases of transfusion transmitted secondary vCJD. However, similar reports of extended incubation periods in *PRNP* codon 129 heterozygous individuals have been described in other acquired forms of human prion disease involving oral and peripheral routes of exposure—specifically, kuru [153] and human growth hormone associated iCJD cases in France [154]. This points to the possibility of further cases of vCJD in this *PRNP* codon 129 genotype.

It is highly likely that UK residents were subject to a substantial dietary exposure to the BSE agent during the 1980s and 1990s, with estimates suggesting that as many as 3 million asymptomatic BSE-infected animals entered the human food chain [12]. However, as of June 2021, the number of clinical cases of vCJD in the UK remains relatively low (n = 178) [80]. Such a discrepancy between the number of vCJD cases reported in the UK and the number of people exposed to the BSE agent raises questions as to the extent of asymptomatic vCJD infection in the UK population. The presence of asymptomatic infection was demonstrated with the detection of PrP^Sc^ and/or infectivity in peripheral tissues of vCJD patients during the long asymptomatic incubation periods [56,91] and following reports of transfusion-transmission of vCJD infectivity from asymptomatic donors who went onto develop vCJD [103].

The observation of PrP^Sc^ in vCJD lymphoreticular tissues, prior to the onset of clinical symptoms, has been utilised in three UK retrospective studies aimed at estimating the prevalence of PrP^Sc^ in the healthy UK population in order to assess the possible risks of further secondary vCJD transmission via blood transfusion and potentially from other medical interventions. In the absence of a blood-based assay, and following the observation of PrP^Sc^ in appendix tissue collected from vCJD patients prior to the onset of disease, analyses of appendectomy (and some tonsilectomy) samples were selected for these studies (Figure 1). The first study (Appendix-I study), reported in 2004, examined PrP immunohistochemical analysis of anonymised appendectomy and tonsillectomy samples [155]. The samples selected for this study had been resected from patients’ post-1995 to allow a greater chance of PrP accumulation in the tissues following the potential exposure of these patients to BSE. The individuals selected were all aged 20–29 years (birth cohort 1961–1985), which was thought to represent the population at greatest risk of developing vCJD. Results confirmed immunostaining consistent with PrP^Sc^ in 3/12,674 samples examined. All three positive samples were found in appendix tissue. These results provided an estimated prevalence of asymptomatic vCJD infection in the UK of 237 per million, or 1 in 4000 of the population, with a wide confidence interval. A second and much larger study (Appendix-II study, n = 32,441) examined appendectomy samples from a wider birth cohort (1940–1981). In this study, 16 positive samples were identified, suggesting an even greater vCJD prevalence of 493 per million of the population, or 1 per 2000, again with a wide confidence interval [156]. Of additional concern from these two studies was the subsequent confirmation that the positive appendix samples originated from patients of all three *PRNP* codon 129 genotypes [156,157]. Importantly, this information supported the findings of animal studies that have shown that all *PRNP* codon 129 genotypes are susceptible to vCJD infection but may be subject to potentially lengthy incubation periods [53].

In view of the elevated prevalence rates indicated by Appendix studies-I and -II, a third study was undertaken (Appendix-III study). In contrast to the previous two studies, Appendix-III examined appendectomy samples from patients who were thought not be exposed to the BSE agent—either having had their appendix removed prior to 1980 and the BSE epidemic (“historical” cases), or having been born after 1996 and the implementation of measures aimed at protecting the human food chain (“new” cases) [158,159]. The purpose of the study was to “test the hypothesis that there would be an absence of samples positive for abnormal PrP in appendices removed from people outside the population considered most at-risk of acquiring vCJD from BSE” [159]. Preliminary results were published by Public Health England in 2016, in which they reported 7/29,516 appendix samples positive for the prion protein [158]. Two of the positive samples originated from the historical cohort of appendices, with the other five originating from the new cohort. The complete data set was published in March 2020 and confirmed a statistically similar prevalence rate to that reported in Appendix-II study [159]. The demonstration of PrP in the appendices collected from individuals thought to have had no exposure to the BSE agent, is difficult to interpret. However, the authors of the study suggest that dietary exposure in the UK population to the BSE agent may have occurred over a more extended time period that initially thought, or that there may be a low prevalence of abnormal PrP in lymphoreticular tissues that does not progress to vCJD.

## 7. Concluding Remarks

Twenty-five years has now passed since the first description of vCJD in the UK. Reassuringly, case numbers in the UK and worldwide remain relatively low with 232 clinical cases of definite or probable vCJD reported to date. Furthermore, following the implementation of rigorous control measures aimed at ensuring food safety, the risk of further dietary exposure of the population to the BSE agent has been almost entirely eliminated. However, as we move forward, uncertainty remains over the potential for future vCJD cases and the enduring implications for public health. The recent emergence of vCJD in a MV heterozygote has raised questions regarding the possibility of a second wave of vCJD in individuals from other codon 129 *PRNP* genotypes (codon 129 MV and VV individuals), perhaps following lengthy incubation periods. Possible changes in the clinical presentation of vCJD in *PRNP* codon 129 MV and VV individuals is uncertain but could impede the identification of further vCJD cases. Furthermore, it is possible that cases of vCJD that occur in older individuals (>65 years) may not be recognised, particularly because other neurodegenerative conditions with similar clinical presentation are relatively common in this age group. Certainly, the current decline observed in the rate of UK hospital autopsies is of great concern in this regard [160].

Following confirmation that vCJD infectivity can be transmitted from human-to-human via transfusion with blood products collected from asymptomatic individuals, the potential for a self-sustained epidemic by blood transfusion is an ongoing public health consideration. Such concerns are exacerbated by the absence of a reliable and rapid blood assay for vCJD. While there is currently no evidence of transmission of vCJD through other medical interventions such as surgery, the potential for such cases to arise cannot be discounted. With such unanswered questions remaining, and the implications for public health, the continuation of surveillance for vCJD and other forms of human prion disease is imperative.
